# Supplementary material for: Genetic Diversity of Grapevine Virus A in Three Australian Vineyards Using Amplicon High Throughput Sequencing (Amplicon-HTS)
Source: Viruses. 2023 Dec 27;16(1):42. doi: 10.3390/v16010042 (PMC10819895; doi:10.3390/v16010042)
Supplement: Supplementary file 1 [file viruses-16-00042-s001.zip › viruses-2726190-supplementary.pdf]

6. Supplementary Materials

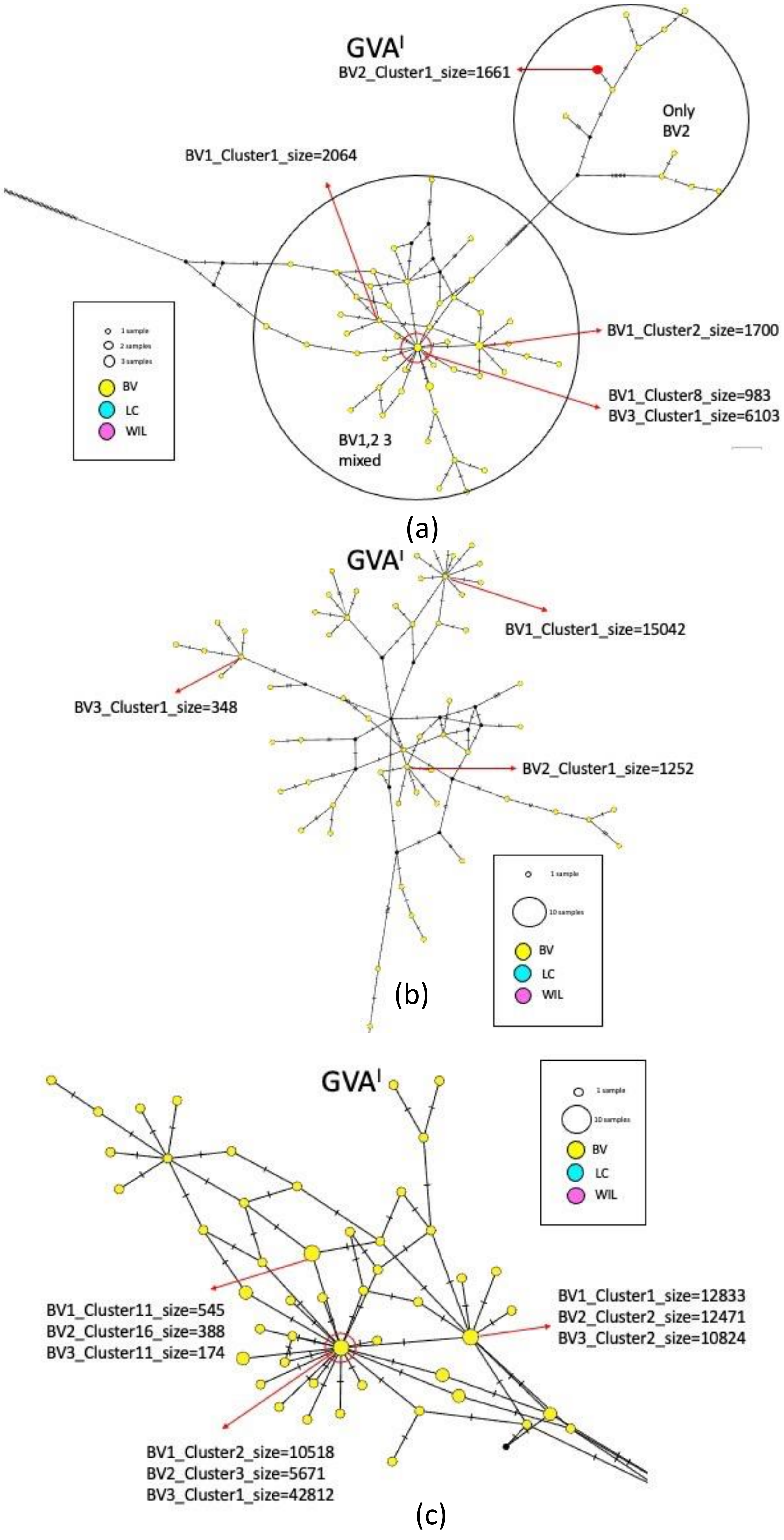

Figure S1. The intra- and inter- host diversity of a total of twenty-three samples affected by shiraz disease (SD) from Langhorne Creek (LC) and Willunga (WIL), and three samples affected by grapevine leafroll disease from Barossa Valley (BV) were analyzed by a phylogenetic median-joining haplotype networks (MJNs) clustering. GVA variants from phylogenetic groups I are labeled as GVA^{I}. Figures (a), (b), and (c) are enlarged MJNs of the coat protein (CP), movement protein (MP) and RNA binding (RB) gene of GVA^{I} variants in Figure 2a, 2b and 2c, respectively

Table S1. The primers used to generate amplicons and the expected amplicon size (base pairs; bp) of the of the grapevine virus A (GVA), coat protein, movement protein and RNA binding protein genes for amplicon high-throughput sequencing (Amplicon-HTS) and for detection of GVA by endpoint RT-PCR.

Virus[1]	Primers	Primer sequence (5′- 3′)	Annealing Temperature (°C)	Virus gene	Amplicon size	Assay	Reference
GVA	Ah587	GACAAATGGCACACTACG	56	Coat protein/ORF 4	430bp	RT-PCR	[55]
	Ac995	AAGCCTGACCTAGTCATCTTGG					
	H7038	AGGTCCACGTTTGCTAAG	56	RNA binding protein/ORF 5	236bp	Amplicon-HTS/RT-PCR	[56]
	C7273	CATCGTCTGAGGTTTCTACTAT					
	GVACP565F	GATACCCTAGTTATGCCAGA	56	coat protein/ORF 4	565bp	Amplicon-HTS	This study
	GVACP565R	CTGTATCACAGTAGCTTCTTG					
	GVAMP404F	CATGGYAAGAATCTGCAAGG	56	movement protein/ORF 3	404bp	Amplicon-HTS	This study
	GVAMP404R	TCTGGCATAACTAGGGTATC					
RubiscoL[1] internal control	RBCL-H535	CTTTCCAAGGCCCGCCTCA	56	N/A	171bp	RNA quality control	[57]
	RBCL-C705	CATCATCTTTGGTAAAATCAAGTCCA					

[1] Internal control primers for RNA quality control. [2] N/A not applicable.

Table S2. The vineyard location, sample ID, sampling time, tissue type, symptom type, variety/clone, year planted of the selected grapevines from three South Australian vineyards, Langhorne Creek (LC), Willunga (WIL) and Barossa Valley (BV) for the amplicon high-throughput sequencing (Amplicon-HTS) experiments.

Location	Sample ID	Tissue type	Sampling time	No. of canes per grapevine	No. of samples	Symptoms	Variety and Clone	Year Established	Grafted on Rootstock?
Langhorne Creek (LC)	LC1 to 15	autumn canes	14/5/2018	3	15	Shiraz disease	Shiraz BVRC12	2004	Chardonnay
Willunga (WIL)	WIL1 to 8	autumn canes	11/5/2018	3	8	Shiraz disease	Shiraz BVRC12	2004	own-rooted
Barossa Valley (BV)	BV1 to 3	autumn canes	09/5/2018	3	3	mild leafroll disease	heritage Shiraz, unknown clone	≈1900 σ	own-rooted

Table S3. Number of amplicon sequencing reads generated for the three gene regions of grapevine virus A.

Gene[1]	Location[2]	Sample ID	Raw reads	Quality trimming: Reads	Quality trimming: % remaining	Size trimming: Reads	Size trimming: % remaining	Primer trimming: Reads	Primer trimming: % remaining	Unique variants after cut-off[a]	Unique aa variants	Nonsense variants	Cluster 1[b]	Cluster 2	Cluster 3	Cluster 4	Cluster 5	Cluster 6	Cluster 7	Cluster 8	Cluster 9	Cluster 10	Cluster 11	Cluster 12	Cluster 13	Cluster 14	Cluster 15	Cluster 16	Cluster 17	Cluster 18	Cluster 19	Cluster 20
		LC1	217,318	101,750	93.64%	84,046	77.35%	81,708	75.20%	524	161	7	22,327	16,980	3,972	547	377	361	320	313	288	285	242	202	189	178	164	159	157	153	151	138
		LC2	248,634	115,371	92.80%	90,658	72.92%	88,525	71.21%	590	381	17	51,254	2,516	757	509	451	438	420	395	275	262	261	247	240	237	236	230	226	213	211	203
MP	LC	LC3	316,538	142,517	90.05%	114,899	72.60%	111,767	70.62%	607	186	10	20,717	8,894	7,121	1,363	1,009	725	621	569	458	416	359	354	339	339	305	304	302	290	280	274
		LC4	253,104	113456	89.65%	96,360	76.14%	93,972	74.26%	515	327	11	46,928	698	537	532	518	486	453	430	417	414	414	363	360	357	351	336	323	317	312	304
		LC5	298,918	139,639	93.43%	111,920	74.88%	109,288	73.12%	678	431	17	63,436	4,058	1,723	1,093	714	497	454	394	320	319	290	290	287	268	264	257	255	253	244	243

		LC6	294,096	137,754	93.68%	112,760	76.68%	110,383	75.07%	644	441	16	63,885	1,267	1,060	763	536	534	534	374	372	370	364	361	356	303	290	288	284	279	267	264
		LC7	250,288	114,222	91.27%	88,521	70.74%	86,495	69.12%	533	348	12	45,608	2,210	582	564	469	401	360	347	318	316	315	294	289	286	285	280	279	258	252	239
		LC8	280,678	125,720	89.58%	103,976	74.09%	100,755	71.79%	621	255	10	30,659	12,751	4,324	3,473	1,122	582	485	446	445	403	392	386	381	363	348	325	304	300	250	246
		LC9	264,532	107,201	81.05%	80,199	60.63%	77,364	58.49%	459	274	12	40,965	1,912	1,299	733	406	322	320	319	299	287	265	256	254	246	231	229	229	229	211	208
		LC10	228,504	99,513	87.10%	77,920	68.20%	75,329	65.93%	464	280	12	40,246	1,554	1,401	520	425	423	335	326	311	304	285	270	264	242	239	230	230	215	212	208
		LC11	233,492	97,875	83.84%	72,045	61.71%	70,079	60.03%	420	259	9	36,645	2,252	698	510	437	330	327	311	302	280	272	269	264	244	229	212	208	199	191	188
		LC12	211,318	88,706	83.95%	70,987	67.19%	69,080	65.38%	409	193	8	28,775	3,007	1,809	881	708	474	320	296	279	270	257	256	252	217	203	195	186	179	178	173
		LC13	246,956	116,953	94.72%	97,214	78.73%	95,120	77.03%	594	340	13	49,376	4,136	3,781	1,096	677	532	514	477	427	424	333	316	298	245	239	235	230	228	224	219
		LC14	252,652	120,654	95.51%	101,089	80.02%	97,904	77.50%	497	224	11	23,976	16,808	1,982	623	568	547	538	538	529	503	485	446	439	419	358	321	290	290	276	218
		LC15	276,306	131,937	95.50%	107,987	78.16%	105,134	76.10%	658	338	16	46,073	1,981	1,512	1,498	1,183	805	639	628	557	545	535	511	480	465	459	456	423	421	416	416
	WIL	WIL1	245,302	109,644	89.40%	91,812	74.86%	89,309	72.82%	596	196	13	25,479	15,350	3,968	530	455	455	440	360	319	271	235	231	222	212	199	195	187	187	179	172
		WIL2	206,068	89,292	86.66%	77,346	75.07%	75,213	73.00%	411	136	10	11,630	8,771	7,294	981	929	824	768	641	403	315	308	270	266	261	252	231	197	196	187	171
		WIL3	274,498	115,968	84.49%	92,163	67.15%	89,952	65.54%	591	325	14	43,101	6,386	4,648	1,116	862	348	264	260	242	240	227	223	204	199	193	190	188	183	173	173
		WIL4	102,720	39,416	76.74%	33,087	64.42%	32,188	62.67%	219	98	7	11,091	4,200	531	411	192	155	110	109	105	102	99	97	94	88	86	85	79	78	77	76
		WIL5	381,238	177,844	93.30%	149,167	78.25%	145,294	76.22%	975	445	29	59,226	2,494	1,821	1,523	1,457	912	892	837	789	772	735	704	670	652	633	617	613	589	572	553
		WIL6	267,442	128,102	95.80%	107,090	80.08%	104,860	78.42%	609	353	14	51,287	2,467	1,337	676	636	556	545	434	366	325	311	299	298	285	284	284	276	273	271	267
		WIL7	205,952	93,487	90.79%	71,846	69.77%	69,905	67.88%	418	218	8	32,063	2,199	1,074	467	465	433	357	346	287	282	253	252	251	241	239	238	238	235	223	220
		WIL8	259,524	124,432	95.89%	106,466	82.05%	104,284	80.37%	600	287	12	42,099	2,423	2,271	1,328	1,279	1,028	945	910	574	556	546	510	482	467	397	392	386	340	332	332
	BV	BV1*	258,588	118,116	91.35%	35,334	27.33%*	33,599	25.99%	238	73	5	15,042	1,237	620	611	271	237	183	180	166	160	148	144	137	127	123	118	118	99	89	88
		BV2*	191,480	82,850	86.54%	9,750	10.18%*	8,838	9.23%	88	15	0	1,252	800	457	244	200	109	91	90	85	73	71	68	63	61	50	47	45	43	40	38
		BV3*	199,966	86,706	86.72%	3,509	3.51%*	2,439	2.44%	25	9	0	348	224	222	130	83	48	45	28	26	26	25	24	21	16	16	16	16	14	13	12
CP	LC	LC1	452,700	207,485	91.67%	134,684	59.50%	131,106	57.92%	761	112	5	9,679	8,968	7,105	4,359	1,345	931	761	664	631	621	461	365	344	322	302	301	278	235	232	222
		LC2	319,480	148,895	93.21%	94,916	59.42%	92,369	57.82%	541	331	21	30,794	789	664	663	614	584	430	413	394	368	347	316	292	271	266	255	237	233	222	216
		LC3	230,566	97,618	84.68%	62,455	54.18%	60,478	52.46%	397	207	10	5,174	2,003	1,842	1,137	241	211	173	151	145	143	141	137	125	115	112	108	107	96	92	87
		LC4	202,170	91,462	90.48%	54,316	53.73%	52,658	52.09%	331	176	9	14,091	2,430	934	248	224	204	198	168	165	161	155	149	142	135	134	134	133	122	119	115
		LC5	315,062	138,800	88.11%	86,195	54.72%	83,575	53.05%	511	231	16	19,090	6,910	4,692	741	635	584	560	267	246	212	201	191	167	155	147	146	141	128	126	122
		LC6	272,106	125,897	92.54%	62,792	46.15%	61,131	44.93%	373	178	13	17,721	3,004	710	562	556	293	228	227	193	188	180	180	162	162	149	146	143	142	136	129
		LC7	350,856	163,058	92.95%	74,709	42.59%	72,494	41.32%	470	184	10	12,477	6,015	4,724	829	363	189	187	184	172	164	154	138	136	126	124	123	122	121	117	116
		LC8	223,674	97,547	87.22%	48,182	43.08%	46,688	41.75%	378	173	9	6,862	2,721	1,291	1,119	796	472	368	278	254	205	169	162	147	138	125	115	104	101	100	83

		LC9	331,944	139,221	83.88%	50,750	30.58%	49,052	29.55%	384	192	19	12,333	1,422	1,333	303	245	234	212	187	181	166	155	152	148	145	144	123	119	117	113	110
		LC10	226,406	98,317	86.85%	67,534	59.66%	65,420	57.79%	369	197	10	19,495	910	806	642	344	327	240	220	210	196	189	187	185	170	167	167	165	161	153	149
		LC11	299,072	127,148	85.03%	62,425	41.75%	60,653	40.56%	362	169	8	15,002	2,417	1,311	783	645	575	547	274	249	210	202	192	189	180	180	178	171	165	157	155
		LC12	321,830	147,381	91.59%	82,281	51.13%	79,772	49.57%	447	231	11	16,805	2,488	1,964	1,077	625	370	360	331	316	270	247	244	244	224	212	191	191	179	179	178
		LC13	332,664	149,085	89.63%	78,468	47.18%	75,308	45.28%	431	183	10	18,803	5,064	1,645	953	715	501	382	361	283	269	262	211	194	189	189	188	180	159	150	143
		LC14	319,244	145,564	91.19%	63,679	39.89%	61,617	38.60%	385	195	11	7,197	5,240	703	687	599	467	417	367	272	268	207	160	149	144	139	138	134	127	125	117
		LC15	378,102	174,410	92.26%	119,104	63.00%	115,705	61.20%	878	411	30	27,349	2,071	1,597	1,052	906	772	638	586	535	482	460	452	446	417	391	383	380	360	339	330
		WIL1	513,022	236,650	92.26%	157,580	61.43%	153,102	59.69%	876	144	8	10,069	9,490	7,673	4,392	1,359	847	751	671	658	597	472	432	355	347	323	267	257	232	230	204
		WIL2	362,178	166,958	92.20%	110,174	60.84%	107,339	59.27%	993	356	39	10,392	5,045	3,543	1,770	1,234	594	484	476	385	353	327	298	280	254	240	233	221	181	173	172
		WIL3	552,274	245,251	88.81%	88,337	31.99%	86,090	31.18%	507	281	12	24,322	2,361	1,040	676	450	372	330	321	274	249	235	212	211	210	205	204	204	191	187	180
	WIL	WIL4	499,358	230,096	92.16%	91,055	36.47%	88,443	35.42%	479	270	15	25,054	1,699	695	503	475	430	346	344	339	332	314	294	257	247	222	222	221	219	216	211
		WIL5	458,846	209,340	91.25%	102,437	44.65%	98,756	43.05%	730	349	35	21,997	540	513	440	419	377	375	372	351	345	327	320	319	305	283	274	271	263	263	255
		WIL6	363,750	157,266	86.47%	78,461	43.14%	76,091	41.84%	422	231	12	22,853	639	561	377	252	237	231	225	213	211	208	199	185	185	181	180	172	171	170	169
		WIL7*	309,620	121,091	78.22%	28,519	18.42%*	27,621	17.84%	271	127	6	2,250	1,380	1,149	937	659	500	414	288	192	187	166	165	117	96	94	92	81	69	67	66
		WIL8	359,502	164,959	91.77%	76,685	42.66%	74,146	41.25%	415	153	9	13,890	3,620	705	676	432	362	354	242	217	210	209	202	190	185	173	170	169	168	160	159
		BV1	274,456	100,920	73.54%	69,344	50.53%	67,264	49.02%	436	57	3	2,064	1,700	1,596	1,533	1,281	1,185	1053	983	706	590	540	539	443	310	305	301	233	215	204	196
	BV	BV2	165,704	56,636	68.36%	37,947	45.80%	35,226	42.52%	178	106	35	1,661	314	305	264	232	222	208	194	193	173	171	171	157	151	143	143	133	124	119	118
		BV3	349,700	136,115	77.85%	97,216	55.60%	93,016	53.20%	778	145	13	6,103	2,411	2,059	1,894	1,745	1,113	790	787	749	684	545	471	443	352	302	297	278	257	217	215
		LC1	383,012	177,332	92.60%	171,930	89.78%	156,691	81.82%	629	270	14	61,140	41,319	8,577	2,652	2,626	1,220	1095	1086	910	791	732	725	692	689	566	544	484	381	343	294
		LC2	308,528	141,902	91.99%	136,070	88.21%	123,590	80.12%	391	287	16	96,292	2,928	1,466	940	773	613	515	403	330	312	297	291	289	274	269	266	264	260	242	229
		LC3	260,144	114,146	87.76%	109,750	84.38%	97,387	74.87%	372	220	10	50,087	11,612	3,046	1,561	816	633	633	571	482	469	455	424	423	404	391	389	358	351	342	341
		LC4	234,768	100,467	85.59%	95,260	81.15%	85,266	72.64%	315	222	11	62,054	2,622	1,477	542	393	379	364	364	361	345	342	341	341	339	337	295	280	275	268	262
		LC5	272,524	107,010	78.53%	98,594	72.36%	90,409	66.35%	333	244	12	75,240	937	419	284	279	248	236	235	223	208	196	187	185	184	175	174	172	170	169	164
RB	LC	LC6	79,678	29,403	73.80%	27,752	69.66%	25,238	63.35%	75	46	1	18,568	1,488	262	173	123	95	91	90	89	82	81	76	76	71	70	68	65	63	60	55
		LC7	290,598	106,667	73.41%	96,409	66.35%	87,173	60.00%	318	188	8	49,556	17,889	706	359	344	312	301	296	282	282	279	257	255	255	249	238	228	219	209	201
		LC8	298,130	104,531	70.12%	95,350	63.97%	86,629	58.11%	289	184	11	36,096	30,127	621	617	599	573	419	326	313	297	297	288	268	239	228	204	193	189	188	178
		LC9	293,006	131,043	89.45%	118,079	80.60%	106,362	72.60%	384	256	16	72,162	9,395	1,630	775	597	495	406	402	382	371	368	359	359	323	314	298	293	287	284	268
		LC10	238,110	104,905	88.11%	94,395	79.29%	86,519	72.67%	332	245	13	67,547	661	608	589	492	336	307	290	278	272	269	263	259	247	245	240	236	232	229	228
		LC11	366,474	164,758	89.92%	151,290	82.57%	136,332	74.40%	469	318	21	95,322	6,843	4,077	1,802	1,235	725	542	538	510	459	456	454	435	427	402	400	361	330	329	328

	LC12	336,544	155,500	92.41%	145,008	86.17%	130,010	77.26%	425	248	16	92,100	5,303	2,225	977	724	575	553	546	543	524	518	513	505	500	491	482	477	449	442	427
	LC13	203,404	95,444	93.85%	88,809	87.32%	81,177	79.82%	315	223	12	58,680	2,312	1,227	1,111	839	799	787	785	578	317	293	240	217	211	180	178	172	172	171	157
	LC14	227,060	107,445	94.64%	100,394	88.43%	89,997	79.27%	322	202	9	37,446	25,135	974	916	723	708	679	439	423	372	369	322	304	294	274	266	264	255	242	241
	LC15	257,202	121,931	94.81%	117,084	91.04%	104,092	80.94%	406	271	15	71,000	1,860	1,239	1,100	971	704	672	666	643	602	560	550	535	529	528	517	515	487	484	472
WIL	WIL1	590,016	283,539	96.11%	275,497	93.39%	249,904	84.71%	898	335	19	92,490	66,582	13,020	4,356	4,247	1,918	1779	1457	1391	1359	1252	1244	1147	1035	991	901	888	851	739	666
	WIL2	432,906	206,852	95.56%	199,999	92.40%	176,931	81.74%	569	377	23	112,436	7,737	3,624	1,961	1,467	1,144	1051	1015	1001	964	927	912	903	853	852	831	830	808	777	725
	WIL3	426,814	197,090	92.35%	172,363	80.77%	154,382	72.34%	420	304	20	122,451	1,566	1,029	930	740	533	513	487	478	474	463	451	439	438	431	391	368	366	337	335
	WIL4	406216	193,012	95.03%	185,067	91.12%	144,054	70.92%	408	303	22	109,167	1,631	876	791	758	740	665	657	651	606	593	579	574	557	532	501	490	473	465	462
	WIL5	284,328	131,658	92.61%	126,101	88.70%	108,169	76.09%	456	283	17	68,656	2,414	2,185	1,688	1,151	895	893	888	855	772	764	754	741	668	629	593	563	546	533	525
	WIL6	280,500	131,380	93.68%	122,836	87.58%	112,001	79.86%	368	275	16	86,798	1,348	624	467	433	433	402	378	369	366	363	345	343	334	320	316	305	302	285	278
	WIL7	262,628	119,850	91.27%	108,352	82.51%	98,163	74.75%	398	245	12	57,561	10,414	4,668	1,861	1,006	614	489	436	366	361	349	307	295	293	259	257	250	245	238	235
	WIL8	223,044	97,899	87.78%	89,383	80.15%	80,615	72.29%	329	235	13	56,399	1,928	746	660	618	614	532	460	416	415	410	402	372	370	361	335	330	318	317	313
BV	BV1	154,174	71,287	92.48%	59,808	77.59%	53,314	69.16%	212	80	2	12,833	10,518	6,018	3,270	1,633	1,392	1051	740	672	656	545	383	338	337	308	291	279	240	216	200
	BV2	244,886	108,919	88.95%	88,650	72.40%	78,582	64.18%	313	141	6	25,081	12,471	5,671	2,411	2,077	2,041	1715	1570	1167	790	768	714	674	595	449	388	361	322	287	279
	BV3	213,784	95,242	89.10%	79,056	73.96%	71,659	67.04%	306	190	8	42,812	10,824	1,696	799	354	273	257	235	228	204	174	166	164	154	147	145	130	130	127	121

[1] MP = movement protein, CP = coat protein, RB = RNA- binding protein of grapevine virus. [2] WIL = Willunga, LC = Langhorne Creek, BV = Barossa Valley. All grapevines listed are var. Shiraz. *Samples with a low percentage of remaining reads after the size trimming. [a] Amplicon reads with the frequency of less than 10 were excluded. [b] Original copy numbers of each amplicon variant before clustering.

Table S4. Number of trimmed reads, unique variants, and the lowest percentage pairwise identity within each sample.

Gene	Sample ID	Trimmed reads	Number of nucleotide (nt) variants	Number of amino acid (aa) variants	Lowest pairwise nt identity	Lowest aa identity	No. nt difference	No. aa difference	Phylogroup in this sample	Copy numbers of largest variant cluster	Phylo-group of the largest variant cluster
MP[1]	LC1	81,708	524	161	92.86	96.69	26	4	II	22,327	II
	LC2	88,525	590	381	93.96	96.69	22	4	II	51,254	II
	LC3	111,767	607	186	90.66	92.56	34	9	II	20,717	II
	LC4	93,972	515	327	91.48	94.21	31	7	II	46,928	II
	LC5	109,288	678	431	98.35	96.69	6	4	II	63,436	II
	LC6	110,383	644	441	98.9	97.52	4	3	II	63,885	II
	LC7	86,495	533	348	95.6	97.52	16	3	II	45,608	II
	LC8	100,755	621	255	90.11	92.56	36	9	II	30,659	II
	LC9	77,364	459	274	98.9	97.52	4	3	II	40,965	II
	LC10	75,329	464	280	98.63	97.52	5	3	II	40,246	II
	LC11	70,079	420	259	95.6	97.52	16	3	II	36,645	II
	LC12	69,080	409	193	91.21	93.39	32	8	II	28,775	II
	LC13	95,120	594	340	98.63	97.52	5	3	II	49,374	II
	LC14	97,904	497	224	90.38	92.56	35	9	II	23,976	II

	LC15	105,134	658	338	98.9	96.69	4	4	II	46,073	II
	WIL1	89,309	596	196	92.58	96.69	27	4	II	25,479	II
	WIL2	75,213	411	136	98.08	95.87	7	5	II	11,630	II
	WIL3	89,952	591	325	98.9	96.69	4	4	II	43,101	II
	WIL4	32,188	219	98	98.9	96.69	4	4	II	11,091	II
	WIL5	145,294	975	445	91.21	94.21	32	7	II	59,226	II
	WIL6	104,860	609	353	98.35	96.69	6	4	II	51,287	II
	WIL7	69,905	418	218	98.35	96.69	6	4	II	32,063	II
	WIL8	104,284	600	287	90.38	91.74	35	10	II	42,099	II
	BV1	33,599	238	73	96.15	96.69	14	4	I	15,042	I
	BV2	8,838	88	15	96.15	95.87	14	5	I	1,252	I
	BV3	2,439	25	9	96.43	95.87	13	5	I	348	I
CP[1]	LC1	131,106	761	112	95.27	98.15	23	3	II	9,679	II
	LC2	92,369	541	331	95.68	97.53	21	4	II	30,794	II
	LC3	60,478	397	207	94.24	96.3	28	6	II	5,174	II
	LC4	52,658	331	176	94.86	96.91	25	5	II	14,091	II
	LC5	83,575	511	231	98.77	97.53	6	4	II	19,090	II
	LC6	61,131	373	178	99.18	98.15	4	3	II	17,721	II
	LC7	72,494	470	184	97.94	96.91	10	5	II	12,477	II
	LC8	46,688	378	173	94.65	96.3	26	6	II	6,862	II
	LC9	49,052	384	192	98.35	96.3	8	6	II	12,333	II
	LC10	65,420	369	197	98.97	97.53	5	4	II	19,495	II
	LC11	60,653	362	169	98.97	96.91	5	5	II	15,002	II
	LC12	79,772	447	231	94.44	96.3	27	6	II	16,805	II
	LC13	75,308	431	183	98.97	98.15	5	3	II	18,803	II
	LC14	61,617	385	195	94.86	96.3	25	6	II	7,197	II
	LC15	115,705	878	411	95.47	96.3	22	6	II	27,349	II
	WIL1	153,102	876	144	95.27	97.53	23	4	II	10,069	II
	WIL2	107,339	993	356	$75.72^{II\&III*}$, 91.36^{II*}, 90.74^{III*}	$78.4^{II\&III}$, 88.82^{II}, 90.12^{III}	$118^{II\&III}$, 42^{II}, 45^{III}	$35^{II\&III}$, 18^{II}, 16^{III}	II&III	10,392	II
	WIL3	86,090	507	281	$76.13^{II\&III}$, 90.95^{II}, 88.07^{III}	$79.01^{II\&III}$, 98.77^{II}, 90.74^{III}	$116^{II\&III}$, 44^{II}, 58^{III}	$34^{II\&III}$, 2^{II}, 15^{III}	II&III	24,322	II
	WIL4	88,443	479	270	99.18	96.91	4	5	II	25,054	II
	WIL5	98,756	730	349	97.74	95.06	11	8	II	21,997	II
	WIL6	76,091	422	231	99.18	97.53	4	4	II	22,853	II
	WIL7	27,621	271	127	93.62	95.06	31	8	II	2,250	II
	WIL8	74,146	415	153	$76.13^{II\&III}$, 90.74^{II}, 90.95^{III}	$79.63^{II\&III}$, 89.51^{II}, 90.74^{III}	$116^{II\&III}$, 45^{II}, 44^{III}	$33^{II\&III}$, 17^{II}, 15^{III}	II&III	13,890	II
	BV1	67,264	436	57	97.53	98.15	12	3	I	2,064	I
	BV2	35,226	178	106	93	93.83	34	10	I	1,661	I
	BV3	93,016	778	145	97.12	97.53	14	4	I	6,103	I
RB[1]	LC1	156,691	629	270	94.95	93.94	10	4	I&II[2]	61,140	I&II
	LC2	123,590	391	287	96.46	93.94	7	4	I&II	96,292	I&II
	LC3	97,387	372	220	91.41	93.94	17	4	I&II	50,087	I&II
	LC4	85,266	315	222	97.98	93.94	4	4	I&II	62,054	I&II

LC5	90,409	333	244	98.99	96.97	2	2	I&II	75,240	I&II
LC6	25,238	75	46	97.98	95.45	4	3	I&II	18,568	I&II
LC7	87,173	318	188	94.95	95.45	10	3	I&II	49,556	I&II
LC8	86,629	289	184	94.44	93.94	11	4	I&II	36,096	I&II
LC9	106,362	384	256	95.96	93.94	8	4	I&II	72,162	I&II
LC10	86,519	332	245	94.95	95.45	10	3	I&II	67,547	I&II
LC11	136,332	469	318	95.96	93.94	8	4	I&II	95,322	I&II
LC12	130,010	425	258	97.98	95.45	4	3	I&II	92,100	I&II
LC13	81,177	315	223	97.98	93.94	4	4	I&II	58,680	I&II
LC14	89,997	322	202	95.45	92.42	9	5	I&II	37,446	I&II
LC15	104,092	406	271	95.96	93.94	8	4	I&II	71,000	I&II
WIL1	249,904	898	335	93.94	93.94	12	4	I&II	92,490	I&II
WIL2	176,931	569	377	96.97	93.94	6	4	I&II	112,436	I&II
WIL3	154,382	420	304	95.96	93.94	8	4	I&II	122,451	I&II
WIL4	144,054	408	303	96.46	93.94	7	4	I&II	109,167	I&II
WIL5	108,169	456	283	94.95	92.42	10	5	I&II	68,656	I&II
WIL6	112,001	368	275	97.98	95.45	4	3	I&II	86,798	I&II
WIL7	98,163	398	245	96.97	93.94	6	4	I&II	57,561	I&II
WIL8	80,615	329	235	95.45	93.94	9	4	I&II	56,399	I&II
BV1	53,314	212	80	94.44	93.94	11	4	I&II	12,833	I&II
BV2	78,582	313	141	91.41	90.91	17	6	I&II	25,081	I&II
BV3	71,659	306	190	95.45	93.94	9	4	I&II	42,812	I&II

[1] MP = movement protein, CP = coat protein, RB = RNA binding protein of grapevine virus A. [2] The Phylogenetic relationship of the phylo-group I and II of the RNA-binding gene cannot be differentiated, named "I&II", details described by Wu *et al.* [15]. * $^{II\&III}$ The lowest % nt identities, maximum nt or aa changes calculated using both GVA^{II} and GVA^{III} variants within the current sample, ^{II} The lowest % nt identities, maximum nt or aa changes by GVA^{II} variants; ^{III} The lowest % nt identities, maximum nt or aa changes using only GVA^{III} variants.
